# *Toxoplasma gondii* infection in immunocompromised individuals

**DOI:** 10.1186/1471-2334-13-S1-P4

**Published:** 2013-12-16

**Authors:** Andrei Csep, Sonia Drăghici

**Affiliations:** 1 University of Oradea, Romania

## Background

Toxoplasmosis is a parasitic zoonosis, caused by protozoan *Toxoplasma gondii*.

## Method

Using the CLIA (chemiluminescence immunoassay) method we determined the IgM and IgG *Toxoplasma gondii* antibodies, and by the EIA (enzyme immunoassay) method the IgA *Toxoplasma* antibodies in HIV infected persons in Bihor County, during the year 2012.

## Results

The study included 30 patients, with HIV infection, 26 (86.7%) of them with antiretroviral treatment, while 4 (13.3%) of them without treatment. Two patients (6.66%) without antiretroviral treatment were found with protective antibody for *Toxoplasma gondii*, while 8 (26.66%) patients with antiretroviral treatment were found with the same antibodies. Two cases from the last category (6.66%) were diagnosed with acute forms of the disease, in which we found IgM to IgG toxoplasma seroconversion.

The seroprevalence of *Toxoplasma gondii* antibodies in HIV infected patients was 33.3%.

## Conclusions

The obtained value (33.3%) in our study is approximately similar to results reported on seroprevalence of *Toxoplasma gondii* antibodies in researches with similar purpose. This high percentage proves that *Toxoplasma gondii* is an additional risk factor for immunosuppressed patients.

